# An Italian Retrospective Survey on Bone Metastasis in Melanoma: Impact of Immunotherapy and Radiotherapy on Survival

**DOI:** 10.3389/fonc.2020.01652

**Published:** 2020-09-15

**Authors:** Francesco Mannavola, Mario Mandala, Annalisa Todisco, Vanna Chiarion Sileni, Marco Palla, Alessandro Marco Minisini, Laura Pala, Francesca Morgese, Lorenza Di Guardo, Luigia Stefania Stucci, Michele Guida, Alice Indini, Pietro Quaglino, Virginia Ferraresi, Riccardo Marconcini, Maria Chiara Tronconi, Ernesto Rossi, Olga Nigro, Marcella Occelli, Alessio Cortellini, Silvia Quadrini, Giuseppe Palmieri, Jacopo Pigozzo, Paolo Antonio Ascierto, Maria Grazia Vitale, Sabino Strippoli, Pier Francesco Ferrucci, Rossana Berardi, Giovanni Randon, Pietro Cardone, Giovanni Schinzari, Franco Silvestris, Marco Tucci

**Affiliations:** ^1^Department of Biomedical Sciences and Human Oncology, University of Bari Aldo Moro, Bari, Italy; ^2^Medical Oncology Unit, Department of Oncology and Hematology, Azienda Ospedaliera Papa Giovanni XXIII Hospital, Bergamo, Italy; ^3^Melanoma Oncology Unit, Veneto Institute of Oncology, Scientific Institute for Research, Hospitalization and Healthcare, Padua, Italy; ^4^Melanoma, Cancer Immunotherapy and Development Therapeutics, Istituto Nazionale Tumori IRCCS Fondazione Pascale, Naples, Italy; ^5^Department of Oncology, Azienda Sanitaria Universitaria del Friuli Centrale, Udine, Italy; ^6^Division of Melanoma, Sarcoma and Rare Tumors, European Institute of Oncology, Scientific Institute for Research, Hospitalization and Healthcare, Milan, Italy; ^7^Oncology Clinic, Università Politecnica delle Marche, Ancona, Italy; ^8^Melanoma Medical Oncology Unit, Department of Medical Oncology and Hematology, National Institute of Tumori, Milan, Italy; ^9^IRCCS Giovanni Paolo II, Cancer Institute, Bari, Italy; ^10^Department of Medical Sciences, Dermatologic Clinic, University of Turin, Turin, Italy; ^11^First Division of Medical Oncology, IRCCS Regina Elena National Cancer Institute, Rome, Italy; ^12^Medical Oncology Department, Santa Chiara Hospital, University of Pisa, Pisa, Italy; ^13^Medical Oncology and Hematology Unit, Humanitas Cancer Center, Humanitas Clinical and Research Center, IRCCS, Rozzano, Italy; ^14^Medical Oncology, Fondazione Policlinico Universitario ‘Agostino Gemelli’ IRCCS, Rome, Italy; ^15^Medical Oncology, ASST-Sette Laghi, Ospedale di Circolo e Fondazione Macchi, Varese, Italy; ^16^Medical Oncology Unit, Santa Croce and Carle Teaching Hospital, Cuneo, Italy; ^17^Department of Biotechnological and Applied Clinical Sciences, San Salvatore Hospital, University of L’Aquila, L’Aquila, Italy; ^18^Medical Oncology Unit, Azienda Sanitaria Locale Frosinone, Frosinone, Italy; ^19^Unit of Cancer Genetics, Institute of Genetic and Biomedical Research, National Research Council, Sassari, Italy

**Keywords:** melanoma, bone metastases, SREs, immunotherapy, bisphosphonates, denosumab

## Abstract

**Background:**

We performed a multicenter retrospective observational study to investigate the impact of clinical–pathological features and therapeutic strategies on both the complications and survival of patients with bone metastases (BMs) from malignant melanoma.

**Patients and Methods:**

A total of 305 patients with melanoma and radiological evidence of BMs were retrospectively enrolled from 19 Italian centers. All patients received conventional treatments in accordance with each own treating physician’s practice. Both univariate and multivariate models were used to explore the impact of melanoma features, including skeletal-related events (SREs), and different treatments on both overall survival (OS) and time-to-SREs. The chi-squared test evaluated the suitability of several parameters to predict the occurrence of SREs.

**Results:**

Eighty-three percent of patients had metachronous BMs. The prevalent (90%) bone metastatic site was the spine, while 45% had involvement of the appendicular skeleton. Forty-seven percent experienced at least one SRE, including palliative radiotherapy (RT) in 37% of cases. No melanoma-associated factor was predictive of the development of SREs, although patients receiving early treatment with bone-targeted agents showed 62% lower risk and delayed time of SRE occurrence. Median OS from the diagnosis of bone metastasis was 10.7 months. The multivariate analysis revealed as independent prognostic factors the number of BMs, number of metastatic organs, baseline lactate dehydrogenase levels, and treatment with targeted therapy or immunotherapy. Subgroup analyses showed the best OS (median = 16.5 months) in the subset of patients receiving both immunotherapy and palliative RT.

**Conclusion:**

Based on our results, patients undergoing immunotherapy and palliative RT showed an OS benefit suggestive of a possible additive effect. The apparent protective role of bone targeting agent use on SREs observed in our analysis should deserve prospective evaluation.

## Introduction

Innovative therapies have improved the survival of patients with unresectable metastatic cutaneous melanoma (CM). However, the prognosis remains poor in those harboring negative prognostic factors, such as poor performance status (PS), high tumor burden, brain metastases, and high baseline levels of lactate dehydrogenase (LDH) (1, 2). Also, the genomic landscape of melanoma influences the clinical evolution as well as innate and acquired drug resistance, thus representing an up-growing field of interest (3).

The impact of bone disease (BD) in melanoma has been scarcely investigated. Data from clinical trials indicate that the skeleton is the fourth site of metastasis after lung, liver, and brain, that occurs in about 11–18% of patients (4, 5). Bone metastases (BMs) generate typical skeletal-related events (SREs), such as severe bone pain, pathological fractures, spinal cord compression, hypercalcemia, and need for radiotherapy (nRT) or surgery to the bone, and are common in breast, prostate, and lung cancers while being so far a clinical challenge in other malignancies like melanoma (6). Moreover, the potential beneficial effect of novel anti-melanoma agents on BMs and SREs is, at present, unclear (7).

Data collected from the SEER (Surveillance, Epidemiology and End Results) database and large series from individual medical centers demonstrated that BMs from CM are frequently observed in young patients and are associated with elevated LDH, while the prognosis is apparently poor and almost similar to that of patients developing brain or liver metastases (8). In addition, BMs occur in patients with CM, while they are rarely detected in those with mucosal, uveal, and acral melanoma. Furthermore, BMs are frequently revealed at diagnosis of the metastatic disease as involving single or multiple sites and frequently affect the axial skeleton.

The pathogenesis of BMs is regulated by the interplay among malignant cells, osteoclasts, osteoblasts, and immune cells. Epithelial tumor cells, indeed, produce high RANK-L (the receptor activator of nuclear factor-kB ligand) levels that engulf the bone metastatic sites, thus interfering with osteoclasts through the RANK receptor that is also expressed by cancer cells like melanoma and regulated by interferon (IFN)-γ (9). Systemic agents for the treatment of BMs include inhibitors of bone resorption and anabolic signals, namely, bone-targeting agents (BTAs), aimed at restoring the physiological bone turnover that results profoundly impaired in patients with skeletal colonization. Their use in breast, prostate, and lung cancers is currently well supported in various clinical settings (10), while their effective contribution in patients with BMs from melanoma is still debated. Recent studies reported the synergistic effect of RANK-L blockade combined with immune checkpoint inhibition in patients with BMs from melanoma, suggesting a potential clinical benefit from this strategy (11). Based on preliminary evidence (12, 13), clinical trials are currently ongoing and are aimed at exploring the overall therapeutic effect of denosumab with immunotherapy (NCT-03161756; EudraCT-2016-001925-15).

Growing interest in melanoma has also been oriented in combining an immune checkpoint inhibitor (ICI) with either systemic or local treatment to maximize the antitumor response. Ongoing clinical trials, for example, are exploring the association of targeted therapy and immunotherapy (14–16). They were supported by preclinical and translational studies showing that BRAF and MEK inhibition has immune-modulating effects that increase tumor T cell infiltration as well as tumor antigen exposition and PD-L1 expression (17, 18). Another attractive scenario is combining immunotherapy with radiotherapy (RT), the latter being often used in the case of BMs. The rationale is that tumor irradiation induces cell death, provoking local release of tumor-derived antigens that, in turn, promote T cell cross-priming by dendritic cells and long-term immunological memory (19). This priming of the immune system by RT can synergize with immunotherapy. Consequently, trials evaluating combinations of immunotherapy and RT have progressively increased in the last years, both in melanoma and other malignancies (20).

Herein, we completed a retrospective multicentric survey in an Italian melanoma population bearing BMs to investigate the potential impact of clinical–pathological features and the therapeutic strategies on survival.

## Patients and Methods

### Study Population

In this observational multicenter study, we retrospectively enrolled 305 patients with a diagnosis of melanoma and radiological evidence of BD. All patients received standard treatments in accordance with each own treating physician’s practice in 19 Italian Centers from November 1984 to March 2019. Clinical data were collected throughout the disease course and included features of melanoma, BM detection, access to systemic treatments (immunotherapy, target therapy, or chemotherapy), nRT, as well as date of first SRE and death. When available, other variables, including age, sex, melanoma primary site, histological parameters (e.g., histotype, Breslow, ulceration, number of mitoses, lymphocyte infiltrate, and nodal stage), BRAF/NRAS status, LDH levels, calcemia, time to appearance of BMs, presence of extraosseous metastases, and SRE type, were assessed. Specific information about the systemic treatments (e.g., drug sequences, objective responses, or time of disease progression) were not considered since these are out of the scope of this survey. All patients had written informed consent for clinical data collection and use for research purposes according to each own center’s rules. Ethical approval was not required for this study according to local legislation.

### Statistical Analysis

Descriptive statistics were used for patient demographics and incidence of SREs. The chi-squared test analyzed the relationship of SRE occurrence with the clinical and pathological features of melanoma patients. Survival analyses were completed by the Kaplan–Meier method. Factors for analyses were identified *a priori* and included baseline clinical features and known prognostic factors for metastatic melanoma or BD. The univariate analysis (log-rank test) explored the clinical variables as prognostic factors for overall survival (OS) and time to SREs from BM diagnosis. Patients who did not experience a SRE were censored at the date of death or at the last follow-up visit for the analysis of time to develop SRE. All significant variables in the univariate model were used to build the multivariate analysis of survival using the Cox proportional hazards regression. Patients that were lost in follow-up were not considered for survival analyses. The statistics were completed with the Medcalc software (version 12.7.0.0). A *p* value < 0.05 was considered significant.

## Results

### Baseline Demographic Features

In total, 305 patients with cutaneous (*n* = 290), mucosal (*n* = 6), and uveal (*n* = 9) melanoma were enrolled in the study. The basal clinical and pathological features of the population are described in Table 1. Of note is that the median age at diagnosis was 56 years and 63.3% were male (*n* = 193). A BRAF mutation was documented in 59%, while NRAS mutation was present in 36% of patients. Almost 22% (*n* = 23/105) of patients had LDH levels >2 times the upper limit of the normal range (ULN) at the time of melanoma diagnosis.

**TABLE 1:** Patient demographics and basal melanoma characteristics in the study population.

Characteristics	Frequency	Percentage
Age at melanoma diagnosis (*n* = 305)		
Median, 56 (range = 18–86) years		
**Sex (*n* = 305)**		
Male	193	63.3
Female	112	36.7
**Primary site (*n* = 305)**		
Limbs	82	26.9
Head and neck	39	12.8
Trunk	134	43.9
Occult	35	11.5
Mucosa	6	2.0
Uvea	9	3.0
**Histology (*n* = 239)**		
SSM	88	36.8
Nodular	95	39.7
Acral	9	3.8
Lentigo Maligna	1	0.4
Mucosal	6	2.5
Uveal	9	3.8
Other	31	13.0
**Breslow depth (*n* = 239)**		
≤1 mm	28	11.7
>1–2 mm	48	20.1
>2–4 mm	73	30.5
>4 mm	90	37.7
**Ulceration (*n* = 239)**		
Present	138	57.7
Absent	101	42.3
**Number of mitosis (*n* = 201)**		
≤1	46	22.9
>1	155	77.1
**Tumor-infiltrating lymphocytes (*n* = 169)**		
Brisk	37	21.9
Absent or not brisk	132	78.1
**Lymph node mts (*n* = 263)**		
N0	133	50.6
N+	130	49.4
**BRAF status (*n* = 285)**		
Mutated	168	58.9
Wild type	117	41.1
**NRAS status (*n* = 59)**		
Mutated	21	35.6
Wild type	38	64.4
**LDH levels (*n* = 105)**		
≤2 × ULN	82	78.1
>2 × ULN	23	21.9

*LDH, lactate dehydrogenase; ULN, upper limit of normal; SSM, superficial spreading melanoma; mts, metastases.*

The majority of patients (97%) had extraosseous metastases, which included 247 patients (81%) with more than three metastatic sites and 73 patients (24%) with brain metastases. The onset of BMs and the diagnosis of primary melanoma were mostly metachronous (83%). The prevalent bone metastatic site was the spine (90%), while only 45% of patients had involvement of the appendicular skeleton, including 10% of them with both axial and appendicular colonization. The majority of patients (61%, *n* = 183/301) showed less than five BMs, while 39% (*n* = 118) harbored more lesions. Calcium levels at the time of BM diagnosis were usually normal, whereas 37% of patients (*n* = 95/254) expressed LDH levels > 2 times the ULN.

Almost half of the patients received BTAs, including bisphosphonates (40.8%, *n* = 119/292) or denosumab (6.8%, *n* = 20). With regard to systemic treatments, 33% of patients (*n* = 96/291) received only targeted therapy, 39% (*n* = 114) only ICIs, and 20.6% (*n* = 60) both ICIs and targeted agents, whereas a minority (7%, *n* = 21) underwent only chemotherapy (CHT). Data are summarized in Table 2.

**TABLE 2:** Characteristics of metastatic disease in the study population.

Characteristics	Frequency	Percentage
**Number of metastatic organs (*n* = 305)**		
<3	58	19.0
≥3	247	81.0
**Presence of extraosseous mts (*n* = 305)**		
No	9	3.0
Yes, without brain	223	73.1
Yes, with brain	73	23.9
**Time of BM diagnosis (*n* = 305)**		
Synchronous	50	16.4
Metachronous	255	83.6
**Sites of BM (*n* = 300)**		
Axial	165	55
Appendicular	29	9.7
Both	106	35.3
**Number of BM (*n* = 301)**		
<5	183	60.8
≥5	118	39.2
**Calcaemia at BM diagnosis (*n* = 235)**		
≤ULN	218	92.8
>ULN	17	7.2
**LDH levels at BM diagnosis (*n* = 254)**		
≤2 × ULN	159	62.6
>2 × ULN	95	37.4
**Use of BTA (*n* = 292)**		
No	153	52.4
Bisphosphonates	119	40.8
Denosumab	20	6.8
**Systemic treatment (*n* = 291)**		
Targeted therapy	96	33.0
Immunotherapy	114	39.2
Targeted and ICIs	60	20.6
Chemotherapy	21	7.2

*BTA, bone-targeting agents; BM, bone metastases; ICIs, immune checkpoint inhibitors; mts; metastases; LDH, lactate dehydrogenase; ULN, upper limit of normal.*

### Bone Disease and Development of SREs

As shown in Supplementary Figure S1A, 47% (*n* = 137/291) of patients developed at least one SRE, which included nRT in 37% (*n* = 109), fractures in 12% (*n* = 35), spinal cord injury in 7% (*n* = 21), and surgery in 3% (*n* = 8), while hypercalcemia occurred in a single case (< 1%). Thirty-four patients (12%) experienced two or more SREs.

The next set of analyses (Table 3) explored the features of melanoma patients who experienced SREs. The median age was 59 years and 65% were male. The majority of them showed metachronous BMs (87%) located in the axial skeleton (56%) as well as LDH levels ≤ 2 times the ULN (67%) and normal calcemia (89%) at the time of BM detection. Notably, 66% of patients had less than five BMs, while extraosseous metastases occurred in about 95% of patients. Among patients who experienced at least one SRE, almost 54% were previously treated with BTAs such as bisphosphonates.

**TABLE 3:** Patient demographics and BM characteristics in the population who experienced SREs.

Characteristics	Frequency	Percentage
**Age at BM diagnosis (*n* = 137)**		
**Median, 59 years**		
**Sex (*n* = 137)**		
Male	89	65.0
Female	48	35.0
**BRAF status (*n* = 131)**		
Mutated	71	54.2
Wild type	60	45.8
**NRAS status (*n* = 27)**		
Mutated	11	40.7
Wild type	16	59.3
**LDH levels at BM diagnosis (*n* = 109)**		
≤2 × ULN	73	67.0
>2 × ULN	36	33.0
**Calcaemia at BM diagnosis (*n* = 104)**		
≤ ULN	93	89.4
> ULN	11	10.6
**BM and melanoma diagnosis (*n* = 137)**		
Synchronous	18	13.1
Metachronous	119	86.9
**SRE and BM diagnosis (*n* = 129)**		
Synchronous	38	29.5
Metachronous	91	70.5
**Localization of BM (*n* = 133)**		
Axial	74	55.6
Appendicular	17	12.8
Both	42	31.6
**Number of BM (*n* = 134)**		
<5	89	66.4
≥5	45	33.6
**Presence of extraosseous mts (*n* = 137)**		
No	7	5.1
Yes	130	94.9
**Use of BTA (*n* = 137)**		
No	63	46
Bisphosphonates	67	48.9
Denosumab	7	5.1

*BM, bone metastases; BTA, bone-targeting agents; LDH, lactate dehydrogenase; mts, metastases; SRE, skeletal-related events; ULN, upper limit of normal.*

Neither clinical parameters nor tumor-related variables correlated with SREs (data not shown). However, patients who were early treated with BTAs showed a minor SRE occurrence with respect to the untreated patients [20% vs. 39.6%; odds ratio (OR) = 0.38, 95% confidence interval (CI) = 0.2–0.72, *p* = 0.003] (Supplementary Figure S1B). Moreover, the univariate analysis (Table 4) investigated those factors putatively associated with longer time to SRE development and revealed a correlation with the axial localization of BMs [hazard ratio (HR) = 0.61, 95% CI = 0.33–1.13, *p* = 0.05] and with previous use of BTAs (HR = 0.41, 95% CI = 0.26–0.66, *p* = 0.001). However, only the use of BTAs before the development of SREs was confirmed as an independent prognostic factor (*p* = 0.003).

**TABLE 4:** Univariate and multivariate analyses of factors associated with time to SRE in patients with BM from melanoma.

Factors	Effect tested	Univariate analysis			Multivariate analysis		
			
		HR	95% CI	*p*	HR	95% CI	*p*
**Demographics**							
Age (years)	≤55 vs. >55	1.22	0.86–1.74	0.25			
Sex	Male vs. Female	1.13	0.80–1.61	0.48			
**Baseline melanoma characteristics**							
Histology	SSM vs. Nodular	0.96	0.61–1.50	0.85^a^			
	Mucosal vs. Nodular	0.95	0.23–3.94				
	Acral vs. Nodular	1.42	0.47–4.34				
	Uveal vs. Nodular	0.50	0.17–1.43				
	Others vs. Nodular	0.81	0.34–1.93				
BRAF genotype	V600 vs. Wild type	0.74	0.52–1.07	0.10			
NRAS genotype	Mutated vs. Wild type	1.37	0.65–2.90	0.33			
**Characteristics present at BM diagnosis**							
Time of diagnosis	Synchronous vs. Metachronous	0.75	0.47–1.20	0.27			
Localization of BM	Axial vs. Extra-axial	0.61	0.33–1.13	**0.05**	0.73	0.36–1.48	0.39
Number of BM	<5 vs. ≥5	0.98	0.68–1.41	0.91			
Calcaemia	≤ULN vs. >ULN	0.70	0.28–1.72	0.35			
LDH levels	≤2× ULN vs. > 2× ULN	1.00	0.66–1.52	1.00			
**Treatment and SRE**							
Use of BTA before SRE	Yes vs. No	0.41	0.26–0.66	**0.001**	0.43	0.24–0.75	**0.003**
Systemic treatment	Targeted/ICIs vs. CHT	1.12	0.51–2.44	0.78			

*BM, bone metastases; BTA, bone-targeting agents; CHT, chemotherapy; ICIs, immune checkpoint inhibitors; LDH, lactate dehydrogenase; SSM, superficial spreading melanoma; SRE, skeletal-related events; ULN, upper limit of normal. ^*a*^Logrank test. Bold values are indicate to p < 0.05.*

### Factors Associated With Overall Survival

At the time of data lock, 182 patients were deceased and 108 were still alive, while the other 15 resulted lost in follow-up. The median OS was 10.7 months (Supplementary Figure S2). Table 5 describes data from both univariate and multivariate analyses of the factors potentially correlated with prognosis. The univariate analysis revealed a worse prognosis in males (*p* = 0.03) and in patients with melanoma of the trunk (*p* = 0.05) as well as a number of five or more BMs (*p* < 0.0001), high LDH levels at metastatic BD diagnosis (*p* < 0.0001), and evidence of three or more metastatic sites (*p* = 0.0027), or brain metastasis (*p* = 0.0002). In addition, patients who received new agents (targeted therapy and/or immunotherapy) showed OS improvement with respect to those treated with CHT (*p* < 0.0001). In addition, neither detrimental impact on OS was determined by the occurrence of SREs, nor in the case of multiple SREs (data not shown). The multivariate analysis confirmed as positive strong independent prognostic factors less than five skeletal metastasis (HR = 0.56, 95% CI = 0.39–0.79, *p* = 0.0013; Supplementary Figure S3), limited LDH values (HR = 0.49, 95% CI = 0.34–0.71, *p* = 0.0001), less than three metastatic sites (HR = 0.58, 95% CI = 0.34–0.99, *p* = 0.047), and treatments with targeted agents and/or ICIs (HR = 0.32, 95% CI = 0.17–0.58, *p* = 0.0002).

**TABLE 5:** Univariate and multivariate analyses of baseline factors associated with OS in patients with BM from melanoma.

Factors	Effect tested	Univariate analysis			Multivariate analysis		
			
		HR	95% CI	*p*	HR	95% CI	*p*
**Demographics**							
Age (years)	≤55 vs. >55	0.75	0.56–1.01	0.07			
Sex	Male vs. Female	1.41	1.05–1.89	**0.03**	1.43	0.97–2.10	0.08
**Baseline melanoma characteristics**							
Histology	SSM vs. Nodular	0.61	0.42–0.89				
	Mucosal vs. Nodular	1.62	0.45–5.87				
	Acral vs. Nodular	0.56	0.23–1.34	0.07^*a*^			
	Uveal vs. Nodular	0.95	0.38–2.39				
	Others vs. Nodular	0.79	0.34–1.85				
Melanoma site	Limbs vs. Trunk	0.68	0.47–0.97		0.87	0.56–1.35	0.54
	Others vs. Trunk	1.09	0.70–1.71	**0.05**^*a*^	1.08	0.69–1.69	0.73
	Occult vs. Trunk	0.65	0.41–1.03		0.75	0.41–1.38	0.36
BRAF genotype	V600 vs. Wild type	1.21	0.89–1.64	0.22			
NRAS genotype	Mutated vs. Wild type	0.87	0.48–1.55	0.65			
**Characteristics present at BM diagnosis**							
Time of diagnosis	Synchronous vs. Metachronous	1.08	0.73–1.59	0.68			
Localization of BM	Axial vs. Extra-axial	1.20	0.76–1.89	0.46			
Number of BM	<5 vs. ≥5	0.47	0.34–0.64	**<0.0001**	0.56	0.39–0.79	**0.0013**
Calcaemia	≤ULN vs. >ULN	0.54	0.23–1.26	0.06			
LDH levels	≤2 × ULN vs. >2 × ULN	0.42	0.30–0.60	**<0.0001**	0.49	0.34–0.71	**0.0001**
**Characteristics of metastatic disease**							
Number of metastatic organs	<3 vs. ≥3	0.55	0.40–0.77	**0.0027**	0.58	0.34–0.99	**0.047**
Presence of visceral mts	Yes with Brain vs. Yes w/o Brain	1.79	1.23–2.62	**0.0002^*a*^**	1.33	0.88–2.01	0.18
	No vs. Yes w/o Brain	0.42	0.21–0.87		0.58	0.13–2.71	0.49
**Treatment and SRE**							
Use of BTA	Yes vs. No	0.80	0.60–1.06	0.13			
SRE occurrence	Yes vs. No	0.85	0.64–1.14	0.28			
Systemic treatment	Targeted/ICIs vs. CHT	0.24	0.10–0.57	**<0.0001**	0.32	0.17–0.58	**0.0002**

*BM, bone metastases; BTA, bone targeting agents; CHT, chemotherapy; ICIs, immune checkpoint inhibitors; LDH, lactate dehydrogenase; mts, metastases; SSM, superficial spreading melanoma; SRE, skeletal-related events; ULN, upper limit of normal; w/o, whitout. ^*a*^Logrank test. Bold values are indicate to p < 0.05.*

### Impact of Melanoma-Dedicated Treatments on Overall Survival

**FIGURE 1:**
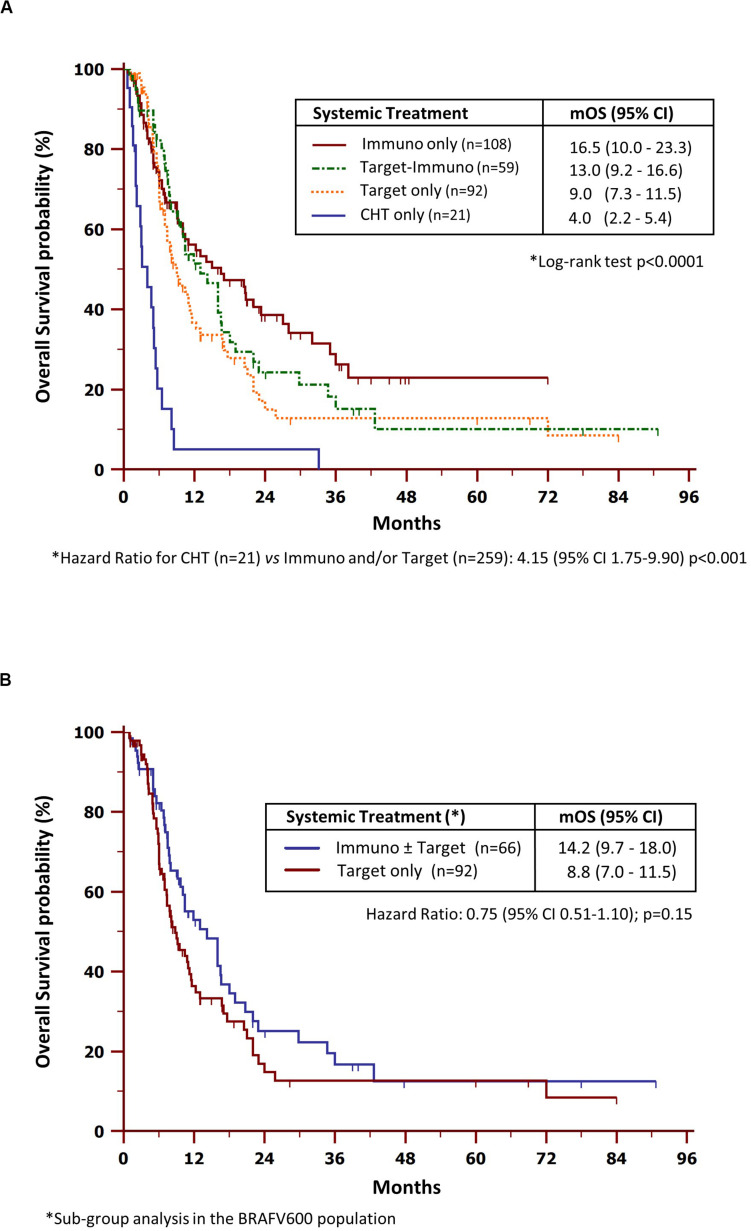
Overall survival by systemic treatment in the study population **(A)** and BRAF-mutated population **(B)**. *mOS*, median overall survival; *CHT*, chemotherapy.

The next set of analyses explored the role of systemic treatments and RT on OS. As shown in Figure 1A, patients receiving CHT alone underwent worsened survival as compared to those treated with new agents, including ICIs and/or targeted drugs (HR = 4.15, CI = 1.75–9.90, *p* < 0.0001). In detail, the median OS (mOS) were 16.5 (95% CI = 10.0–23.3), 13.0 (95% CI = 9.2–16.6), 9.0 (95% CI = 7.3–11.5) and 4.0 months (95% CI = 2.2-5.4) in patients comprehensively treated with (i) ICIs only, (ii) ICIs and targeted therapy, (iii) targeted therapy only, or (iv) CHT only, respectively.

Further investigations were dedicated to the BRAF-mutated population (Figure 1B). The median OS in patients who received at least one ICI (14.2 months, 95% CI = 9.7–18.0) resulted increased with respect to those treated with targeted therapy alone (8.8 months, 95% CI = 7.0–11.5), although the differences were not statistically significant (HR = 0.75, 95% CI = 0.51–1.10, *p* = 0.15).

**FIGURE 2:**
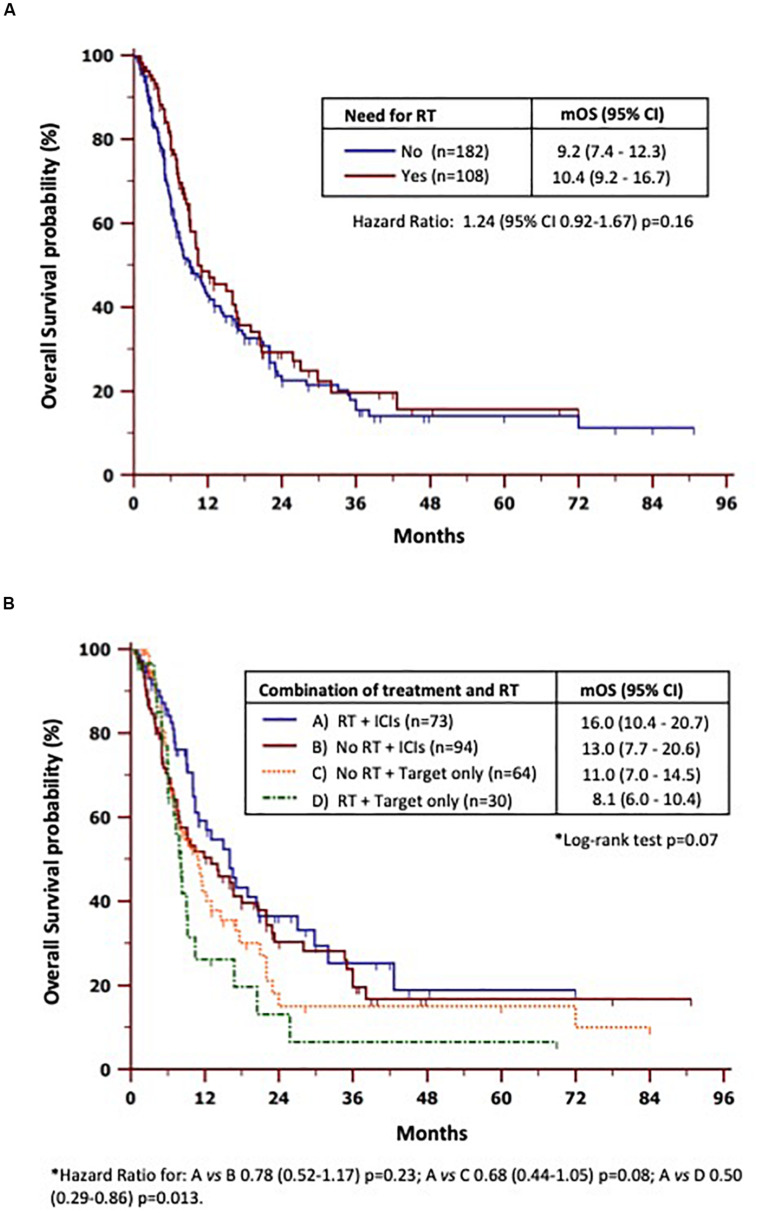
Subset analyses of overall survival by radiotherapy need **(A)** and combinations of radiotherapy and new therapies **(B)**. *mOS*, median overall survival; *RT*, radiotherapy.

Regarding bone-specific treatments (Supplementary Figure S4), patients who received BTAs showed only a modest benefit in terms of mOS as compared to the untreated ones (11 vs. 9 months), but not statistically significant differences were found (HR = 0.80, 95% CI = 0.60–1.06, *p* = 0.13). On the other hand, mOS was quite similar between patients who underwent RT (Figure 2A) vs. those who were never treated (10.4 vs. 9.2 months). We also verified (Figure 2B) whether or not different combinations of RT with new therapies interfered with survival. To this purpose, we divided the study population into four groups based on the treatment received: (A) ICIs and RT (*n* = 73); (B) ICIs without RT (*n* = 94); (C) targeted therapy only without ICIs (*n* = 64); and (D) targeted therapy plus RT and never ICIs (*n* = 30). We found that patients in group A achieved the best mOS (16 months, 95% CI = 10.4–20.7) with respect to either group B (13 months; HR = 0.78, 95% CI = 0.52–1.17, *p* = 0.23), group C (11 months; HR = 0.68, 95% CI = 7.0–14.5, *p* = 0.08), or group D (8.1 months; HR = 0.5, 95% CI = 0.29–0.86, *p* = 0.013).

## Discussion

The development of BMs frequently occurs in cancer with a negative impact on the quality of life and survival. Appropriate algorithms for the management of BD mainly derive from extensive prospective trials with patients harboring tumors that frequently metastasize to the skeleton, such as breast, prostate, lung, and kidney cancers (21). Otherwise, a definite evidence-based strategy for the treatment of BMs from other malignancies endowed with lower osteotropic propensity does not exist, while apparently innovative drugs such as cilengitide failed to provide satisfactory results to be applied for routine clinical practice (22, 23). Particularly, the impact of BD in melanoma has been poorly investigated, as well as the potential therapeutic strategies such as either RT or BTA use. Moreover, another unanswered question from recent registrative clinical trials in melanoma concerns the possible impact of ICIs or targeted agents in patients with BM.

The present study was aimed at exploring retrospectively the characteristics of melanoma patients bearing BD. As a result of the available literature to the topic, ours appears as a large retrospective study investigating the features of BMs in melanoma. The definition of the effective incidence of BMs in melanoma, however, is out of the scope of the study and, therefore, was not investigated.

The baseline demographic data in our melanoma population demonstrated that BMs occurred primarily in males with prevalent involvement of the axial skeleton. Almost all patients (97%) showed extraosseous metastases. Among them, 83% developed metachronous bone and visceral metastases, while in 13% of them BMs were discovered in consequence of a SRE occurrence. The frequency of patients harboring a BRAF mutation (59%) was almost in line with that observed in the general melanoma population. On the other hand, the relative higher frequency observed for mutated NRAS (35.6%) should not be considered as a major propensity of these patients in developing BMs since the NRAS mutational status was available only in less than 20% of cases.

Other studies describing a modest incidence of bone involvement at diagnosis of metastatic melanoma probably reflect the fact that the BD was not properly suspected and investigated at diagnosis. However, one-fourth (23.9%) of melanoma patients from our series showed a brain involvement which is instead reported in about 40–60% of the general melanoma population (24). This apparent difference from our data may probably imply specific molecular mechanisms which critically drive the osteotropism of melanoma cells like those affecting the SDF1/CXCR7/CXCR4 pathway, as recently proven (25, 26).

The median survival from the onset of BM was 10.7 months, but our analysis revealed that patients receiving innovative therapies including ICIs and/or targeted agents showed a better prognosis (9.0–16.5 months) than those undergoing CHT (4.0 months). The patients analyzed in this study (*n* = 290) apparently showed a lower OS with respect to those enrolled in recent phase 3 clinical trials with new agents resulting in 5-year survival rates higher than 50%. However, it is noteworthy that our survey refers to the time from BM diagnosis because the information relative to the time of a comprehensive diagnosis of metastatic disease was not available. The lack of a control group without BMs also restrains the possibility of exploring the real impact of the BD in the general melanoma prognosis. Moreover, our real-world experience includes patients with clinical features that are generally excluded from clinical trials, including a suboptimal PS or brain involvement, thus reducing the possibility of a direct comparison between various case studies.

The data from large clinical trials with recent 5-year survival updates and pooled analyses defined major negative prognostic factors in metastatic melanoma that included elevated LDH levels, poor PS, and three or more metastatic sites. Despite the lack of information regarding the PS of our population, our multivariate model was in line with these results, while revealing for the first time that an elevated number of BM (five or more skeletal lesions) significantly harms the survival. Additionally, the mutational status of melanoma patients bearing BM did not apparently influence OS, although a positive trend was seen in BRAF-mutated patients treated with immunotherapy (14.2 months) as compared to those treated with targeted therapy alone (8.8 months). These results, however, are conditioned by an *a priori* selection bias since the clinician therapeutic decisions were driven by individual prognostic factors; therefore, they should not lead to considering a superiority of immunotherapy in this setting.

SREs are major complications of BMs that restrain the quality of life in cancer patients. The 2-year cumulative incidence of SREs in breast, prostate, and lung tumors ranges from 41 to 54%, and more than one half of patients develop a SRE at cancer diagnosis or thereafter. In the majority of these tumors, SREs dramatically impact on cancer-specific survival (27, 28). Similarly to previous studies (29–31), we observed that at least half of patients with BMs developed a SRE, whose nRT predominantly occurred in 37% of the studied population, while 12% of these patients experienced more than a single SRE. Moreover, in the majority of patients (85%), SREs followed the diagnosis of BMs, with about 50% of patients experiencing one SRE within 1 year from diagnosis.

No melanoma-associated factors were predictive for SRE development in our cohort, although we observed that those receiving primary treatment with BTAs showed a 62% reduced risk of experiencing SREs and delayed time to their occurrence. This apparent protective effect of BTAs in reducing the risk of SREs sounds very impressive if compared to other osteotropic tumors whose relative risk reduction ranges from 15 to 30% (32, 33). However, it is almost difficult to speculate on the possible more potent effect of BTAs in the treatment of melanoma BMs due to the retrospective nature of our study. Finally, a possible effect of BTAs on OS was not demonstrated in our melanoma population. Other studies investigated the efficacy of combining immunotherapy with anti-RANK-L monoclonal antibodies in this setting of patients, providing encouraging though weak results that undoubtedly require further investigation (11). Based on our data, the use of BTAs should be thus suggested at the time of BM detection since it reduces the incidence and delays the development of SREs.

In a different fashion from other tumors, SREs had no impact on the survival of our melanoma patients bearing BMs, including the nRT that was the most recurrent complication. The modest effect of RT on prognosis was previously demonstrated since the majority of patients showed lower survival and required further treatments to stabilize the skeletal complications (34). However, relevant data from our study concerns the protective effect in terms of survival for patients receiving RT and immunotherapy, independently of targeted agents. Therefore, it is conceivable that the OS benefit observed in these patients could have balanced the worse prognosis of those combining RT and targeted therapy without ICIs.

Benefits derived from the combination of RT and immunotherapy have been widely described as abscopal effect in melanoma (35) as well as in other malignancies (36) and reflects the regression of non-irradiated metastatic lesions at a distance from the primary site of irradiation. The putative synergic effect of RT and immunotherapy gained by our population is also suggested by the similar effect of immunotherapy vs. targeted therapy in the absence of RT, although both a defect of enrollment and radiological imaging as well as the retrospective nature of the analysis are probably limiting factors in our study. However, this combined effect also results from restoration of the immune cell activity in their systemic anticancer effect. In addition, it has been demonstrated that T cells may protect against bone loss (37), while IFN-γ counterbalances the osteoclastogenesis by interfering with RANK signaling (38). Finally, osteoblasts are also influenced by local T-helper 2 cells through parathormone (PTH) production (39), and a role of plasmacytoid dendritic cells has also been described (40).

## Conclusion

In conclusion, we identified the number of BM as a novel prognostic factor in metastatic melanoma and observed that both reduced risk and delay in SRE development occur in patients early treated with BTAs. Despite the SREs not impacting on the survival of melanoma patients with BM, those receiving immunotherapy and requiring palliative RT obtained a major extent of benefit in terms of OS. Further prospective studies are thus needed to understand the effective role of immunotherapy in melanoma patients with BD. However, our preliminary observation suggests that palliative RT on symptomatic BMs may potentially reinforce the immune response and T cell activity in patients treated with ICIs, while the complementary activity of BTAs requires further investigation.

## Data Availability Statement

The raw data supporting the conclusions of this article will be made available by the authors, without undue reservation.

## Ethics Statement

Ethical review and approval was not required for the study on human participants in accordance with the local legislation and institutional requirements. The patients/participants provided their written informed consent to participate in this study.

## Author Contributions

MT, MM, VS, MP, AM, LP, FMo, LD, MG, AI, PQ, VF, RM, MCT, ER, ON, MO, AC, SQ, GP, JP, PA, MV, SS, PF, RB, GR, PC, and GS contributed to the provision of study materials or patients. AT did the data collection. FMa contributed to the statistical analysis. FMa and MT did the data interpretation and manuscript writing. All authors are accountable for all aspects of the work, gave final approval of the manuscript, and contributed to revising the manuscript critically.

## Conflict of Interest

The authors declare that the research was conducted in the absence of any commercial or financial relationships that could be construed as a potential conflict of interest.

## Supplementary Material

FIGURE S1 **(A)** Percentage of patients experiencing different skeletal related events (SREs) in the study population. **(B)** Incidence of SREs according to the use of bone-targeted agents (BTAs). nRT, need for radiotherapy; ^∗∗^*p* < 0.01.

FIGURE S2 Kaplan–Meier overall survival estimate in the study population (*n* = 290). mOS, median overall survival.

FIGURE S3 Overall survival by number of bone metastases (BM). mOS, median overall survival; LDH, lactate dehydrogenase.

FIGURE S4 Overall survival according to the use of bone-targeted agents (BTAs) mOS, median overall survival.
