# Supplementary figures and images for: An Italian Retrospective Survey on Bone Metastasis in Melanoma: Impact of Immunotherapy and Radiotherapy on Survival
Source: Front Oncol. 2020 Sep 15;10:1652. doi: 10.3389/fonc.2020.01652 (PMC7523509; doi:10.3389/fonc.2020.01652)

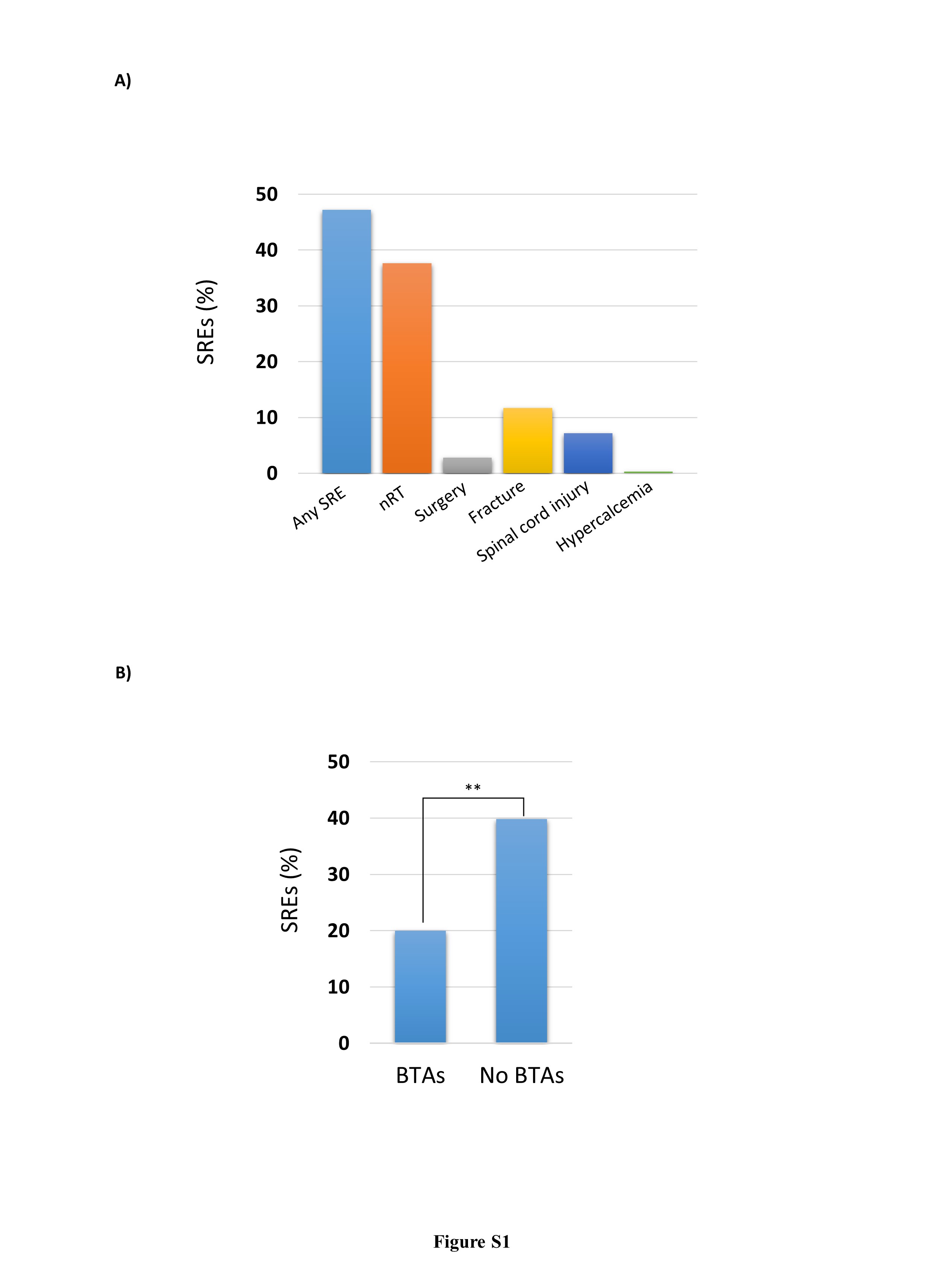

Supplement: FIGURE S1 — (A) Percentage of patients experiencing different skeletal related events (SREs) in the study population. (B) Incidence of SREs according to the use of bone-targeted agents (BTAs). nRT, need for radiotherapy; ∗∗p < 0.01. [file Image_1.JPEG]

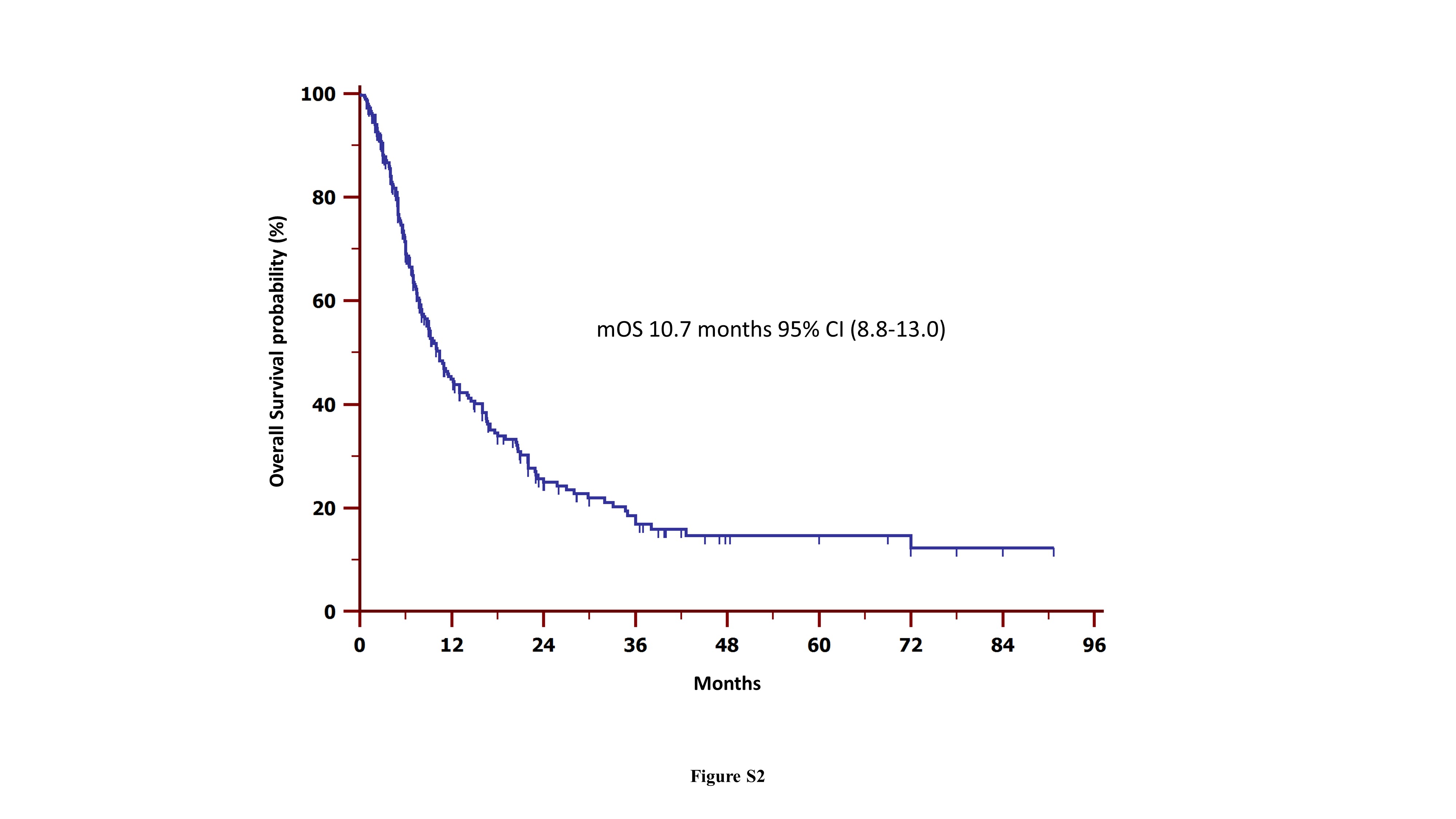

Supplement: FIGURE S2 — Kaplan–Meier overall survival estimate in the study population (n = 290). mOS, median overall survival. [file Image_2.JPEG]

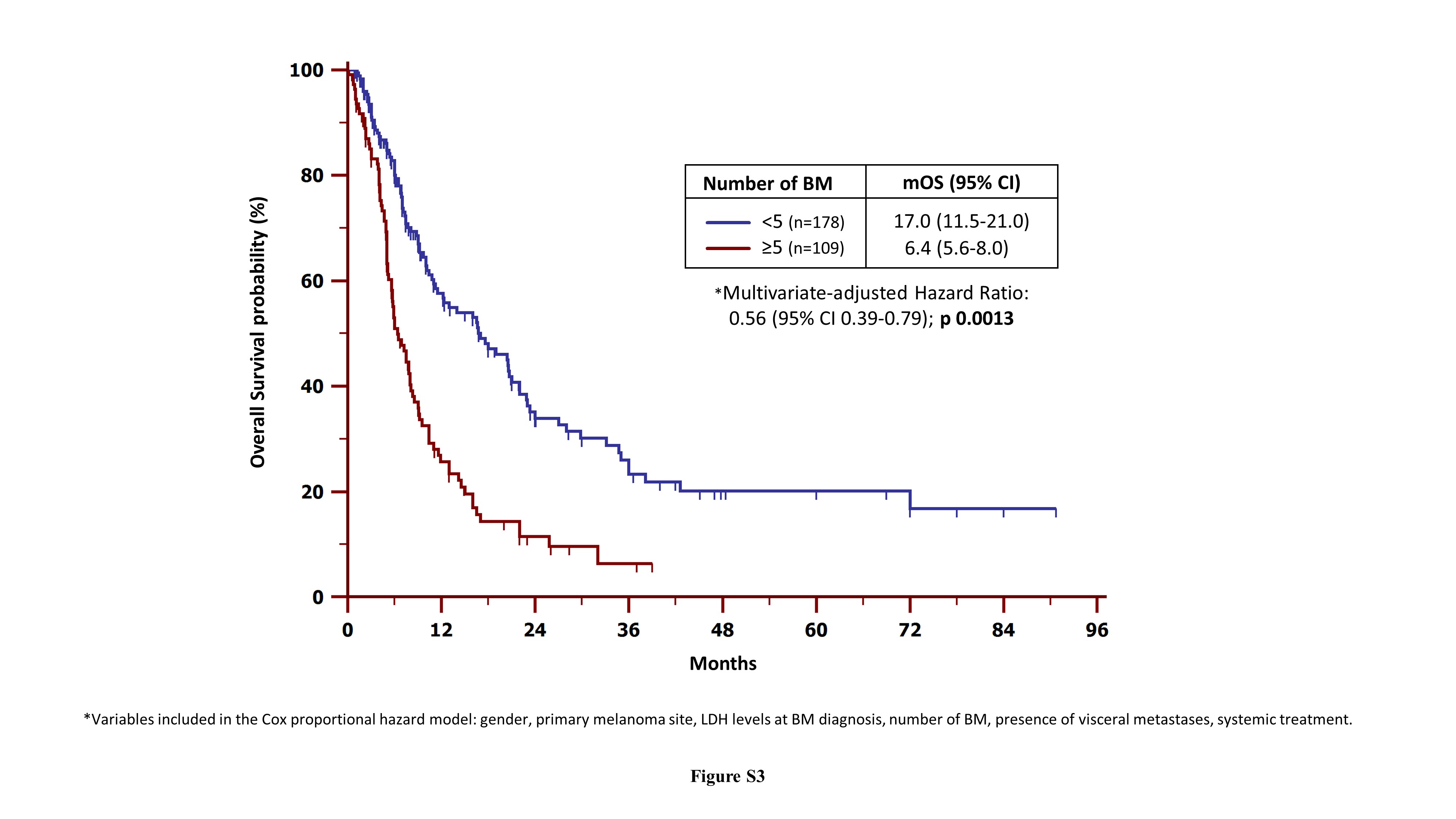

Supplement: FIGURE S3 — Overall survival by number of bone metastases (BM). mOS, median overall survival; LDH, lactate dehydrogenase. [file Image_3.JPEG]

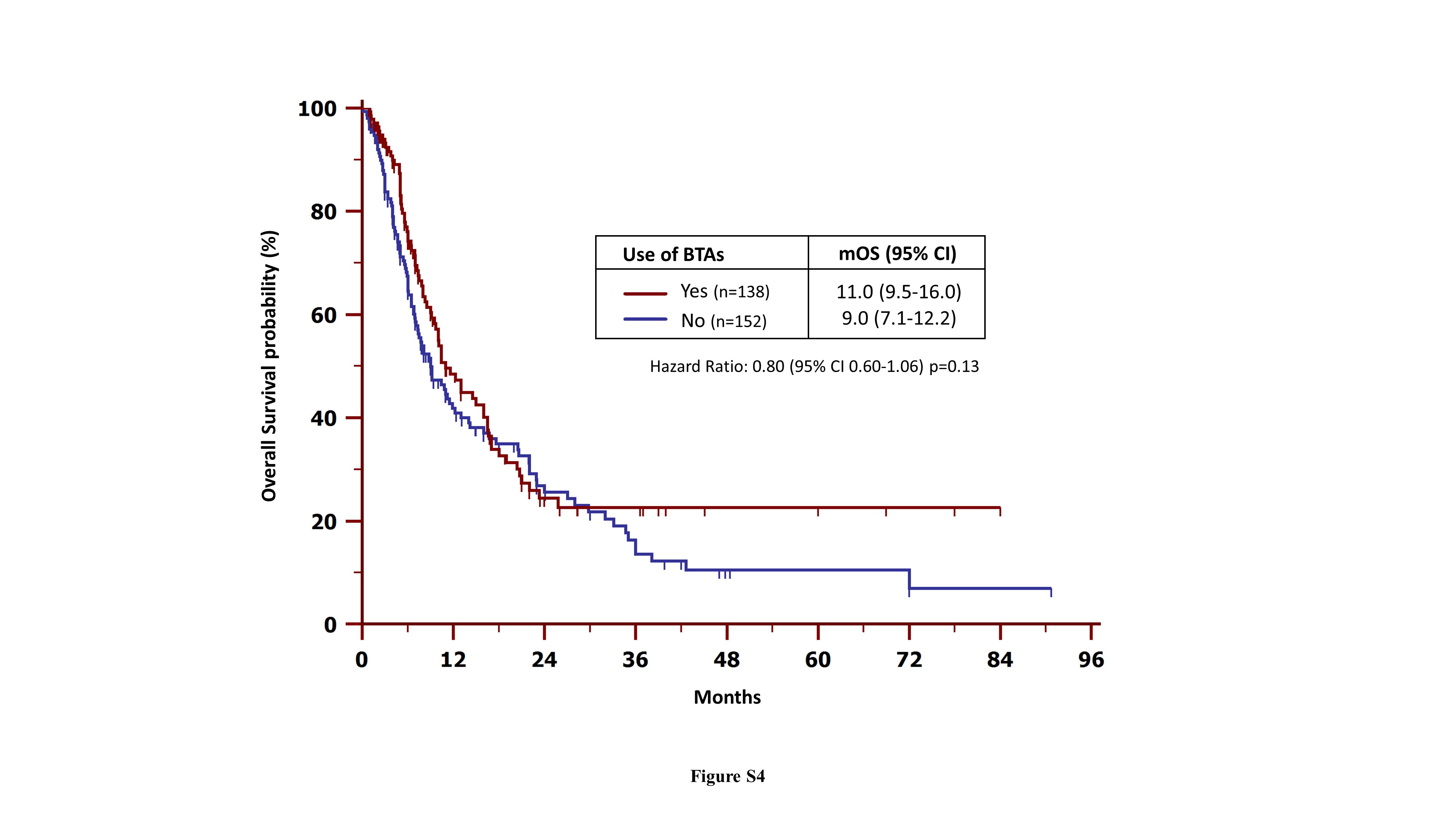

Supplement: FIGURE S4 — Overall survival according to the use of bone-targeted agents (BTAs) mOS, median overall survival. [file Image_4.JPEG]
